# Exploration in association between vitamin D and cutaneous melanoma and explainable machine learning prediction

**DOI:** 10.3389/fonc.2025.1503611

**Published:** 2025-05-08

**Authors:** Lingyi Li, Man Luo

**Affiliations:** ^1^ Outpatient Department Office, The Central Hospital of Wuhan, Tongji Medical College, Huazhong University of Science and Technology, Wuhan, Hubei, China; ^2^ Department of Oncology, Wuhan No.1 Hospital, Wuhan, Hubei, China

**Keywords:** vitamin D, cutaneous melanoma, male, aging, NHANES

## Abstract

**Objective:**

This study aims to examine association between vitamin D with melanoma and develop an explainable machine learning model.

**Methods:**

For this study, relevant data were downloaded from the CDC’s National Health and Nutrition Examination Survey (NHANES) program, for the three survey cycles 2011-2012, 2013–2014 and 2015-2016. Self-reported melanoma data, serum vitamin D levels, and other covariates were downloaded and analyzed. Analysis of variance in this study was performed using t-tests and chi-square tests, modelling was performed using logistic regression based on NHANES weights, and other risk factors were analyzed using forest plots. Ten machine learning models were compared and XGboost was selected for the melanoma prediction.

**Results:**

In this study, logistic regression analysis revealed a protective effect of higher vitamin D levels in melanoma, the ORs were much less than 1 for Q2 (OR=0.97, 95% CI (0.44, 0.98)), Q3 (OR=0.71, 95% CI (0.65, 0.92)), and Q4 (OR=0.32, 95% CI (0.55, 0.81)). Meanwhile, forest plot analysis showed that vitamin D, the number of sunburns in the past year, advanced age, Caucasian, education some college, single and unmarried, smoking, diabetes and hypertension, were all statistically significant. The OR was higher in men than in women, with Q4 values of 0.31 (95% CI: 0.18–0.51) for men and 0.29 (95% CI: 0.15–0.45) for women. OR was higher in the senior patients than in the non-senior group, with Q4 (OR=0.53, 95% CI (0.23, 0.73)). An explainable XGBoost model had AUC 0.906, and in the model vitamin D had main contribution to the model.

**Conclusion:**

In conclusion, this study concluded that vitamin D decreases melanoma risk based on a larger sample and multi-covariate analysis. Female and young people received high protection from vitamin D in melanoma. XGBoost can accurately prediction the possibility of melanoma based on vitamin D.

## Introduction

Over the past 50 years, cutaneous melanoma (CM) incidence has steadily increased around the world. The expected number of new cases of CM in the world in 2018 is 287,723, with an age-standardized incidence rate of 3.1/100,000/year and a mortality rate of 0.63/100,000/year, according to GLOBOCAN 2018 (1, 2). As the most deadly and aggressive type of skin cancer, CM arises from unrepaired DNA damage in skin cells (3, 4). CM is around 5% of skin cancers overall, but it also kills about 75% of people who have skin cancer (5, 6).

In recent years, the potential anti-cancer effects of vitamin D have garnered increasing attention in the field of oncology. Research has linked low vitamin D serum levels to poorer survival rates and increased thickness of CM, supporting the hypothesis that vitamin D derivatives might play a crucial role in cancer prevention and treatment strategies (7, 8). Yet, despite its anti-cancer benefits, vitamin D has also been demonstrated to have immunosuppressive effects, it is not considered to be the standard of therapy (9, 10). A Study have shown that vitamin D can alter the tumor microenvironment by increasing the ratio of T regulatory (Treg) cells to T helper 17 (Th-17) cells, leading to immunosuppression (11). This dual effect necessitates careful consideration when evaluating the role of vitamin D in cancer therapy (12).

Recently machine learning was widely used in disease risk factor exploration and prediction (13, 14). In parallel, the integration of machine learning techniques in melanoma research is transforming the landscape of diagnosis and treatment. Machine learning algorithms can analyze vast datasets, identifying patterns and predictors of CM progression that might not be visible through traditional methods (5). These advanced tools can enhance the accuracy of early detection, predict patient outcomes, and even personalize treatment plans based on individual genetic and environmental factors. By combining machine learning insights with clinical and biochemical data, researchers hope to refine therapeutic strategies and achieve better prognostic accuracy for melanoma patients.

Despite growing evidence on vitamin D’s anti-cancer effects, its role in melanoma remains controversial due to conflicting findings and limited large-scale studies (9, 10). To gain deeper insights into the impact of vitamin D on CM, this study incorporated a large sample size and multiple covariates for model analysis. By doing so, researchers aim to unravel the complex interactions between vitamin D and melanoma and establish effective machine learning prediction model, potentially paving the way for innovative treatment approaches and improved patient outcomes. By integrating epidemiological and computational approaches, this work provides novel insights into personalized prevention strategies.

## Methods

### Data source

**Figure 1:**
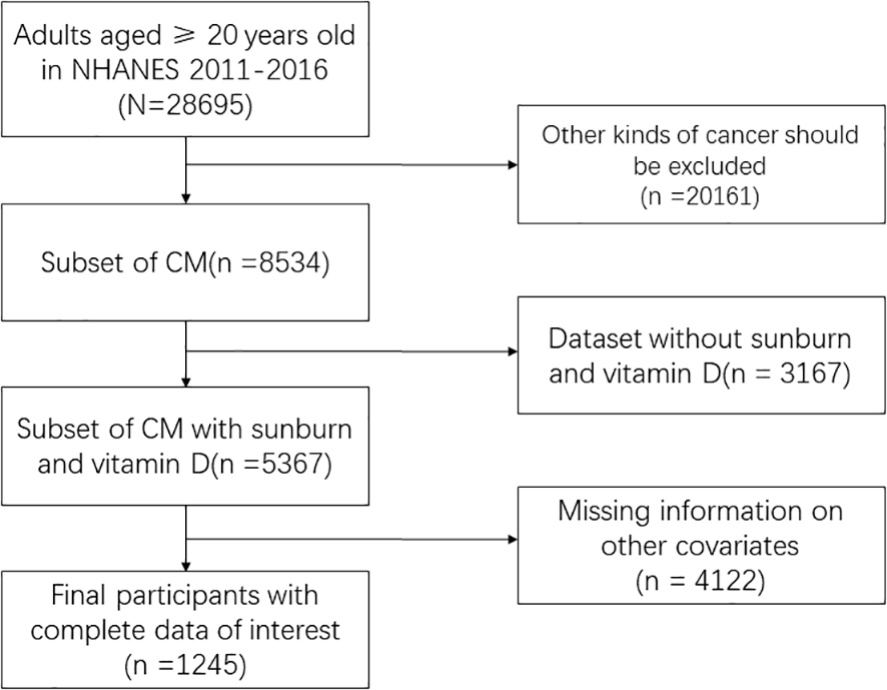
Flow chart of inclusion and exclusion.

The National Health and Nutrition Examination Survey (NHANES) survey data from the CDC, which includes annual dietary nutrition surveys of the US population from 2000 to 2020, served as the source of the data for this study (https://www.cdc.gov/nchs/nhanes/). The data collected included demographic data, data from physical exams, data from lab tests, and data from questionnaires. The required variables were found in three cycles, 2011–2012, 2013–2014, and 2015–2016, which were chosen for this study. A final sample of 1245 people who included all three cycles’ variables was chosen after screening 29250 of the three cycles were included. Finally, we delete the data with missing covariates. The inclusion and exclusion process are shown in the **Figure 1**. This cross-sectional study analyzed data from the CDC’s NHANES program from 2011 to 2016, covering a nationally representative sample of the U.S. population.

### Melanoma and covariates

The NHANES questionnaire’s section on medical conditions asked about “age at first diagnosis of melanoma,” which must be answered in order to be considered a melanoma patient; if not, the respondent is not a melanoma patient. A skin-related questionnaire that revealed the sample’s number of sunburns in the previous year served as the primary source of data for this study’s primary risk factor. In order to measure 25-hydroxyvitamin D3, 3-epi-25-hydroxyvitamin D3, and 25-hydroxyvitamin D2 (25OHD2) in human serum, the US Centers for Disease Control and Prevention (CDC) used high performance liquid chromatography-tandem mass spectrometry (HPLC-MS/MS). The 25OHD3 + 25OHD2 form of vitamin D used in this research.

Other covariates, such as demographic data (including age, sex, race, education, marriage, BMI, and PIR, an indicator of household economic situation), disease history (including diabetes and hypertension), and lifestyle habits (including smoking and alcohol consumption), were obtained from questionnaire data.

### Model development and comparison

The included data were divided into a training set (70%) and an internal validation set (30%) to mitigate overfitting. A prediction model was built using the aforementioned 12 features. Ten ML models, including Decision Tree (DT), K-Nearest Neighbors (KNN), Logistic Regression (LR), Random Forest (RF), Support Vector Machine (SVM), Naive Bayes (NB), GLM with Elastic Net Regularization (GLM), Generalized Additive Model (GAM), Multivariate Adaptive Regression Splines (MARS), and Extreme Gradient Boosting (XGB), were utilized to predict the probability of cutaneous melanoma.

To optimize the prediction models, a combination of grid search and manual tuning was employed to obtain the final hyperparameters. The reliability of the models was evaluated using commonly used metrics such as the area under the ROC curve (AUC), sensitivity, specificity, positive predictive value, negative predictive value, accuracy, and F1 score. Furthermore, 10-fold cross-validation was performed in the derivation cohort to validate the prediction models.

DT offer advantages such as interpretability, ease of visualization, and the ability to handle both numerical and categorical data, but they are prone to overfitting and can be unstable with small data changes. KNN is simple to implement, requires no training phase, and adapts well to local patterns, but it suffers from high computational cost with large datasets and sensitivity to irrelevant features. LR is efficient, interpretable, and works well for binary classification, but it struggles with non-linear relationships and multicollinearity. RF reduces overfitting, handles high-dimensional data, and provides feature importance, but it is less interpretable than single decision trees and can be computationally expensive. SVM excel in high-dimensional spaces, are effective for clear-margin classification, and can use kernels for non-linear data, but they are computationally intensive and require careful parameter tuning. NB is fast, performs well with small datasets, and is ideal for text classification, but it assumes feature independence, which is often unrealistic. GLM combines L1 and L2 regularization for feature selection and multicollinearity handling, offering interpretability, but it may underperform with highly non-linear data. GAM are flexible, handle non-linear relationships, and are interpretable, but they can be computationally expensive and complex to tune. MARS model non-linear and interaction effects effectively and are interpretable, but they can overfit with noisy data and require careful tuning. XGB delivers high performance, handles large datasets, and is robust to outliers, but it is complex to interpret, computationally intensive, and requires extensive hyperparameter tuning. Each model has its trade-offs, making it crucial to select the right one based on the specific problem, dataset size, and interpretability requirements.

### Feature selection and model explanation

Obtaining a correct interpretation of ML models could be challenging. The SHapley Additive exPlanations (SHAP) method was a technique that ranked the importance of input features and explains the predictions of a model. Its implementation aimed to overcome the “black box” problem by assisting in feature selection using SHAP values, which ranked the importance of features. This process helped to reduce the initial 47 features to a final set of 9 features that had the best predictive power. This selected model was then used for further analysis.

The SHAP method provided both global and local explanations for model interpretation. Global explanations provided consistent and accurate attribution values for each feature in the model, illustrating the associations between input features and the progression of kidney function. On the other hand, local explanations demonstrated specific predictions for individual patients by inputting specific data into the model.

### Statistical analysis

First, the baseline data were described according to the prevalence of CM, with all variables in both groups being described in accordance with CM status. Using chi-square tests for categorical variables and t-tests for quantitative data, a variance analysis between the two groups was also carried out.

In order to examine the risk factors impacting CM, stepwise logistic regression analysis was then used to build three models, the first of which had CM with the primary risk factor being serum vitamin D in the previous year. The second model combined CM with information on demographics, lifestyle choices, and the amount of sunburns in the previous year. Moreover, forest patches were made. The third model integrated CM with information on demographics, lifestyle choices, past medical history, and the number of sunburns in the previous year. Forest plots were also used to demonstrate the third model.

A subgroup analysis of CM was then completed, comparing the outcomes of the third model for men and women and for the higher and lower age cases, respectively. The subgroups were divided based on gender and age.

According to the official NHANES weight recommendations, we selected WTMEC2YR as the analytic weight, which was used for both the interviewed and MEC examined sample persons because the majority of the sample data included came from the questionnaire. R4.2.2 (R CORE TEAM, Vienna, Austria) was used to run all analyses for this study, and P 0.05 was regarded as statistically significant.

## Results

The outcomes for each covariate for the groups with and without CM disease are displayed in **Table 1**. As can be seen, only age and the number of sunburns in the previous year showed a statistically significant difference between the two groups. Other factors, including gender, race, education, marital status, PIR, smoking, alcohol use, diabetes, hypertension, and BMI, showed no significant differences.

**Table 1:** Characteristics of included samples.

	Non-CM	CM	P value
	N=1223	N=22	
Sunburn times	1.75 (0.96)	2.29 (2.59)	0.041*
Vitamin D	66.8 (24.6)	67.3 (21.3)	0.965
Age	36.9 (10.9)	50.8 (4.79)	0.01*
Gender			1
Male	616 (50.4%)	11 (50.0%)	
Female	607 (49.6%)	11 (50.0%)	
Race			1
Mexican American	142 (11.6%)	5 (22.72%)	
Other Hispanic	91 (7.44%)	8 (36.36%)	
Non-Hispanic White	764 (62.5%)	4 (18.18%)	
Non-Hispanic Black	77 (6.30%)	2 (9.09%)	
Other Race - Including Multi-Racial	149 (12.2%)	3 (13.64%)	
Education			0.164
Less Than 9th Grade	35 (2.86%)	3 (13.64%)	
9-11th Grade	114 (9.32%)	6 (27.27%)	
High School Grad/GED or Equivalent	254 (20.8%)	4 (18.18%)	
Some college or AA degree	410 (33.5%)	4 (18.18%)	
College Graduate or above	410 (33.5%)	5 (22.72%)	
Marriage status			0.044
Married	606 (49.6%)	11 (50.0%)	
Widowed	12 (0.98%)	2 (9.09%)	
Divorced	111 (9.08%)	2 (9.09%)	
Separated	39 (3.19%)	5 (22.72%)	
Never married	320 (26.2%)	2 (9.09%)	
Living with partner	135 (11.0%)	0 (0.00%)	
PIR	2.73 (1.66)	2.73 (1.68)	0.998
Smoking			0.045
Yes	562 (46.0%)	20 (90.91%)	
No	661 (54.0%)	2 (9.09%)	
Alcohol			0.125
Yes	197 (16.1%)	11 (50.0%)	
No	1026 (83.9%)	11 (50.0%)	
Diabetes			1
Yes	84 (6.87%)	5 (22.72%)	
No	1118 (91.4%)	17 (77.27%)	
Borderline	19 (1.55%)	0 (0.00%)	
Missing	2 (0.16%)	0 (0.00%)	
Hypertension			1
Yes	14 (1.14%)	2 (9.09%)	
No	1209 (98.9%)	20 (90.91%)	
BMI			0.838
normal(25<)	392 (32.1%)	10 (45.46%)	
overweight(25≤BMI<30)	447 (36.5%)	6 (27.27%)	
obesity(≥30)	384 (31.4%)	6 (27.27%)	

*P<0.05.

In model 1, which divides serum vitamin D in the previous year into four quarters, Q3 and Q4 are statistically significant, along with a dose-response relationship, as shown in **Table 2**. Results from Models 2, 3, and 1 are comparable. The vitamin D in the previous year and having CM, however, showed a clear dose-response relationship in Model 4, and Q2-Q4 were all statistically significant when compared to Q1 and all had ORs that were significantly higher than 1. Q2, Q3, and Q4 all had odds ratios of 0.25, 0.33, and 0.32, respectively, with a 95% confidence interval of 0.1 to 0.85.

**Table 2:** Logistic risk analysis for vitamin D and CM.

Sunburn times	Model1	Model2	Model3	Model4
Q1	Reference	Reference	Reference	Reference
Q2	0.92 (0.52, 0.94)	0.63 (0.89, 0.93)	0.74 (0.84, 0.96)	0.97 (0.44, 0.98) *
Q3	0.82 (0.34, 0.83) *	0.32 (0.33, 0.82) *	0.45 (0.73, 0.76) *	0.71 (0.65, 0.92) *
Q4	0.26 (0.10, 0.52) *	0.25 (0.21, 0.45) *	0.33 (0.21, 0.85) *	0.32 (0.55, 0.81) *
p trend	0.03*	0.04*	0.01*	0.01*

*P<0.05, Model 1= Sunburn; Model 2= Model 1 plus Vitamin D; Model 3 = Model 2 plus adjusted for sex, age (years, continuous), age squared, education (less than high school, high school graduate, some college and above), race (non-Hispanic white, non-Hispanic black, Mexican American, other), self-reported alcohol status (Yes and No) and self-reported smoking status (Yes and No); Model 4 = Model 3 plus adjusted for BMI, self-reported hypertension (Yes and No) and self-reported diabetes (Yes and No).

**Figure 2:**
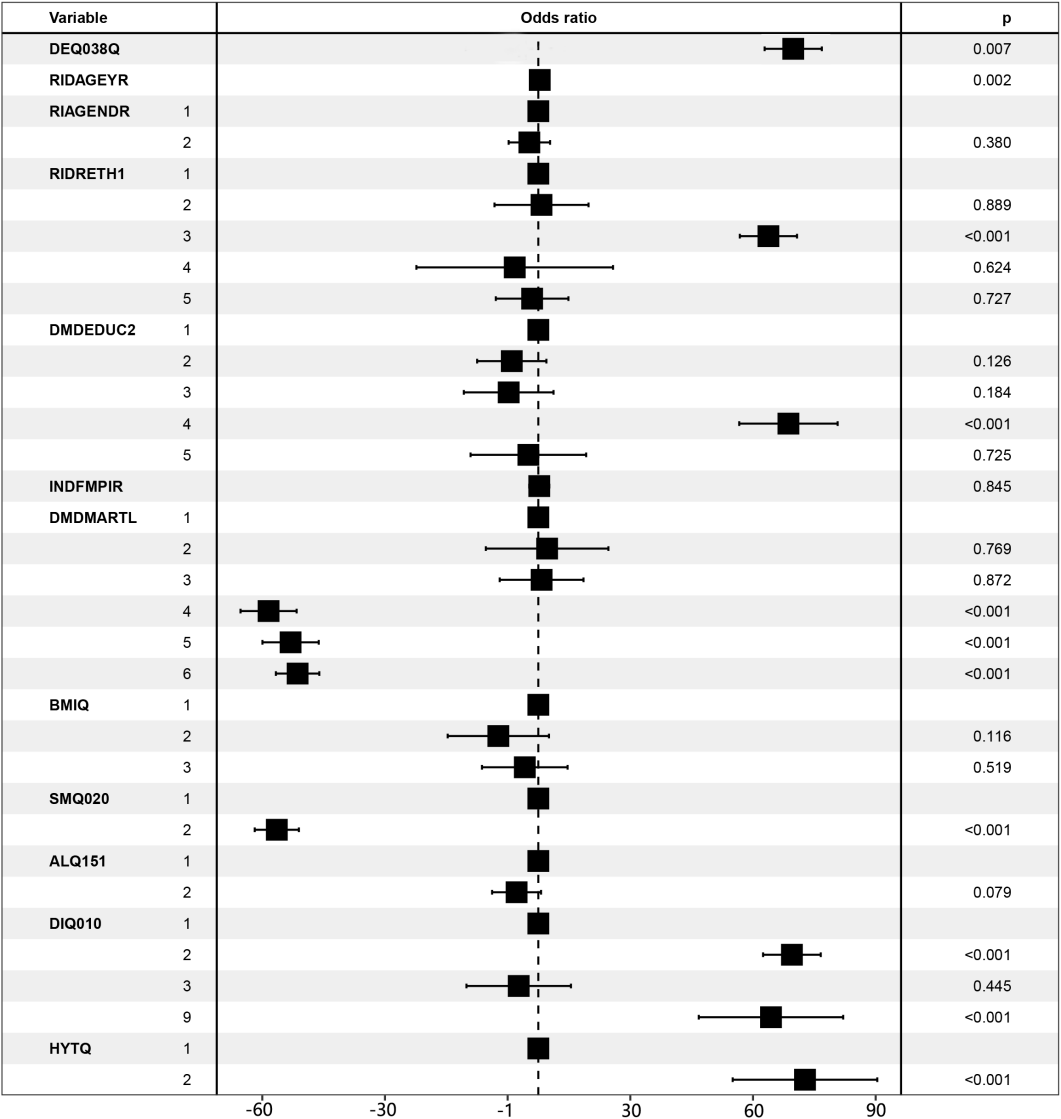
Forest plot for CM risk factors. LBXVIDMS: Serum vitamin D; DEQ038Q: sunburn times; RIDAGEYR: age; RIAGENDR: gender (1=male, 2=female); RIDRETH1: race (1=Mexican American, 2=Other Hispanic, 3=Non-Hispanic White, 4=Non-Hispanic Black, 5=Other Race - Including Multi-Racial), DMDEDUC2: education (1=Less Than 9th Grade, 2 = 9-11th Grade, 3=High School Grad/GED or Equivalent, 4=Some college or AA degree, 5=College Graduate or above); DMDMARTL: marriage (1=Married, 2=Widowed, 3=Divorced, 4=Separated, 5=Never married, 6=Living with partner); BMIQ: BMI (1=normal (25<), 2=overweight (25≤BMI<30), 3=obesity (≥30)); SMQ020: smoking (1= No, 2= Yes); ALQ151: alcohol (1= No, 2= Yes); DIQ010: diabetes (1= No, 2= Yes, 3=Borderline, 9=Missing); HYTQ(1= No, 2= Yes).

We included all covariates in the model and generated forest plots after investigating the relationship between CM and sunburn based on model 3. **Figure 2** demonstrates the statistical significance of the number of sunburns in the previous year, vitamin D, advanced age, Caucasian, some college education, single, unmarried, cohabiting, smoking, diabetes, and hypertension. While some of the aforementioned statistically significant risk factors have been previously reported, others could also be explained by the confounding variables used in this study. It is obvious that additional research into the risk factors for CM is necessary.

### Subgroup analysis

The results of the subgroup analysis are shown in **Table 3**, with statistically significant Q3 (OR=0.64, 95% CI (0.48, 0.81)), Q4 (OR=0.31, 95% CI (0.18, 0.51)) for men, and Q4 (OR=0.29, 95% CI (0.15, 0.45) for women. Men had significantly higher ORs than women did. In the age subgroup, senior patients had higher ORs than non-senior patients, with Q3 having a higher OR in the senior group than the non-senior group (OR=0.74, 95% CI (0.41, 0.98), and Q4 having a higher OR in the senior group than the non-senior group (OR=0.53, 95% CI (0.23, 0.73)).

**Table 3:** Subgroup association between Vitamin D and CM.

Vitamin D	Male	Female	Age<60	Age≥60
Q1	Reference	Reference	Reference	Reference
Q2	0.85 (0.68, 1.87)	0.59 (0.56, 2.98)	0.83 (0.93, 2.53)	0.82 (0.44, 1.56)
Q3	0.64 (0.48, 0.81) *	0.52 (0.17, 1.41)	0.64 (0.43, 0.91) *	0.74 (0.41, 0.98) *
Q4	0.31 (0.18, 0.51) *	0.29 (0.15, 0.45) *	0.49 (0.33, 1.73)	0.53 (0.23, 0.73) *
p trend	0.03*	0.01*	0.02*	0.01*

*P<0.05.

### Explainable machine learning model

**Figure 3:**
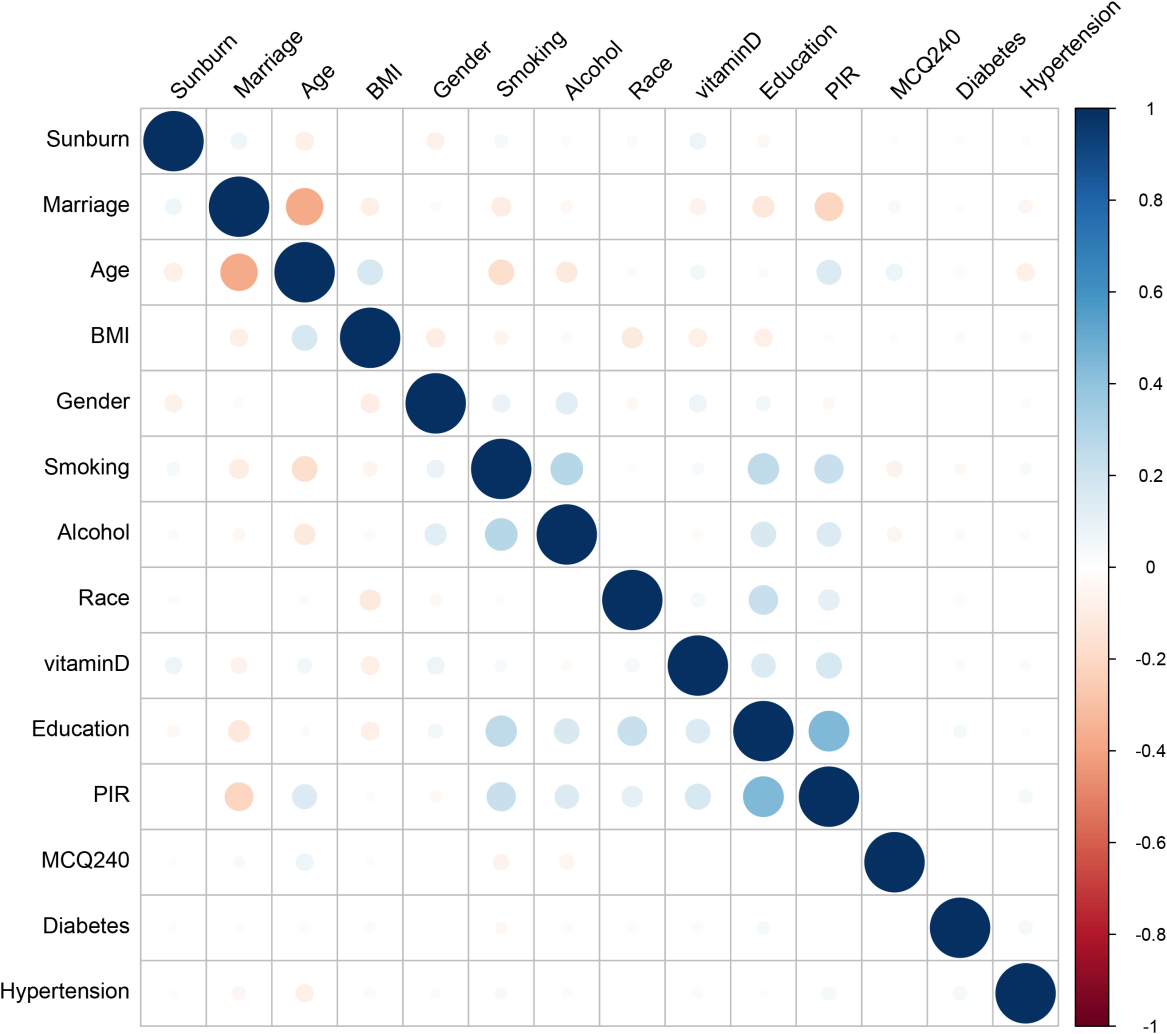
Correlation matrix of included variables.

**Figure 4:**
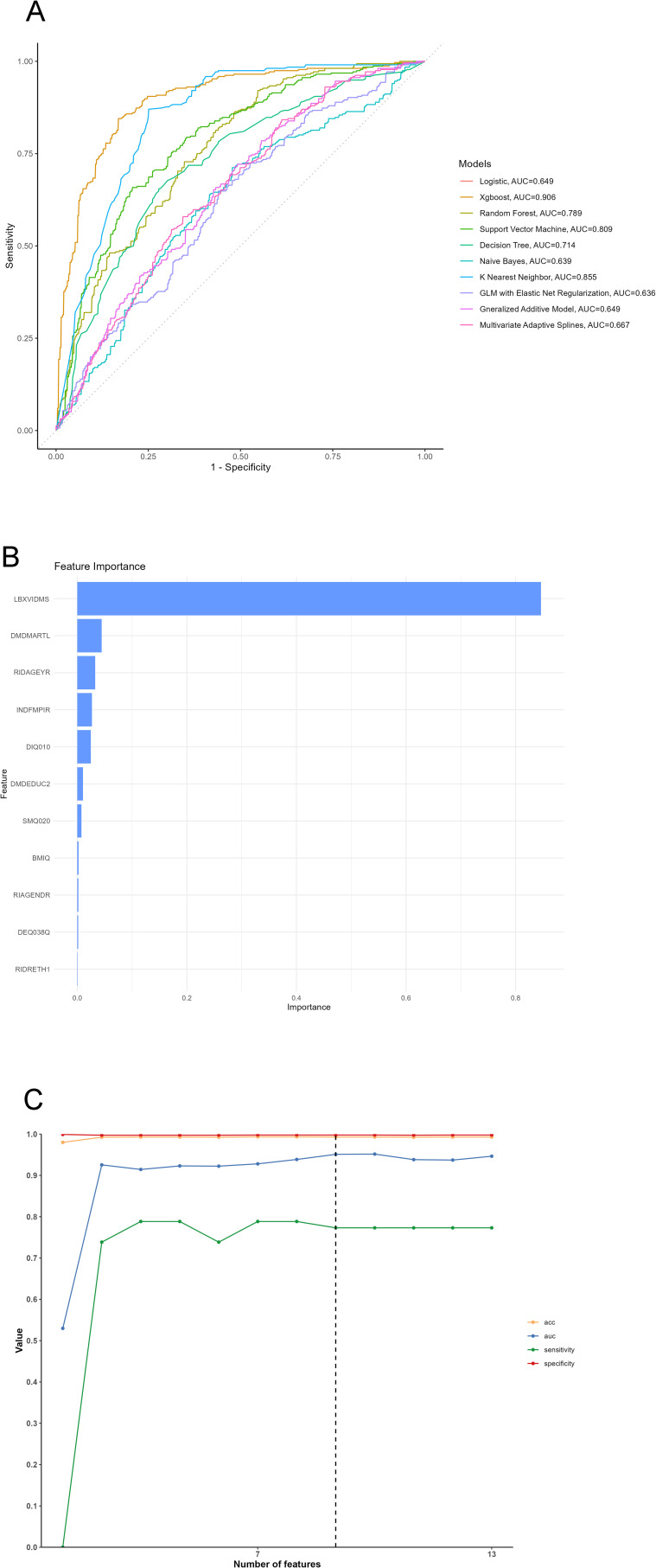
**(A)** ROC Curve of 10 machine learning models, **(B)** Variable importance plot, **(C)** Feature selection processing plot. LBXVIDMS: Serum vitamin D; DEQ038Q: sunburn times; RIDAGEYR: age; RIAGENDR: gender (1=male, 2=female); RIDRETH1: race (1=Mexican American, 2=Other Hispanic, 3=Non-Hispanic White, 4=Non-Hispanic Black, 5=Other Race - Including Multi-Racial), DMDEDUC2: education (1=Less Than 9th Grade, 2 = 9-11th Grade, 3=High School Grad/GED or Equivalent, 4=Some college or AA degree, 5=College Graduate or above); DMDMARTL: marriage (1=Married, 2=Widowed, 3=Divorced, 4=Separated, 5=Never married, 6=Living with partner); BMIQ: BMI (1=normal(25<), 2=overweight (25≤BMI<30), 3=obesity(≥30)); SMQ020: smoking (1= No, 2= Yes); ALQ151: alcohol (1= No, 2= Yes); DIQ010: diabetes (1= No, 2= Yes, 3=Borderline, 9=Missing); HYTQ (1= No, 2= Yes).

The derivation cohort data was used to generate 10 ML models for predicting cutaneous melanoma. Before we conduct the ML training, we made a correlation analysis and it showed that marriage, PIR and Age had negative correlation while education, PIR and smoking had positive correlation (**Figure 3**). Among the 10 models, the XGB model achieved the best predictive performance for “cutaneous melanoma” with an AUC of 0.906. The KNN model (AUC = 0.854) and Support Vector Machine model (AUC = 0.808) followed in terms of performance. The ROC curves for the 9 selected features in all ML models and the AUC summary plot for XGB could be found in **Figure 4A**. Therefore, it could be observed that among the mentioned five models, the XGB model performed the best in predicting “ cutaneous melanoma “. The performance of the XGB model with different numbers of features was shown in **Figure 4C**. Sensitivity, specificity, positive predictive value (PPV), negative predictive value (NPV), accuracy, and F1 score were calculated at the optimal cutoff value that maximized the Youden index. The final model was determined during the feature simplification process of the XGB model. We analyzed the importance of all 45 features, as shown in **Figure 4B**. The 9-feature model demonstrated good net benefit and high threshold probability, as depicted in **Figure 4C**.

### Model explanation

**Figure 5:**
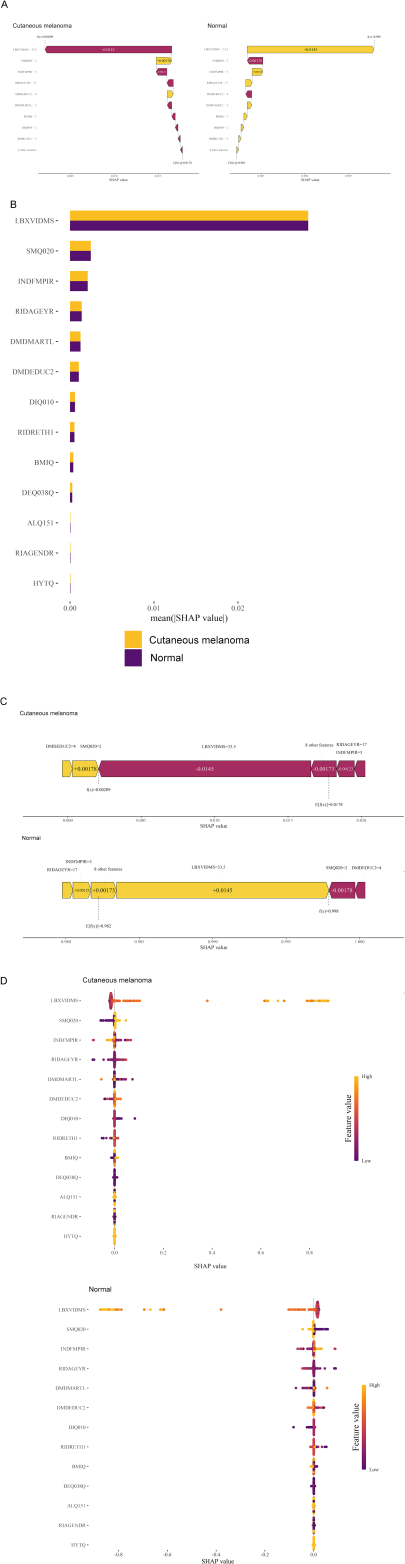
Global model explanation by the SHAP method. **(A)** Waterfall plot and evolution of risks contributed by each feature for individual patient at high risk of cutaneous melanoma and low risk **(B)** SHAP summary bar plot. **(C)** Force plot for the internal validation set at high risk of cutaneous melanoma and low risk. **(D)** SHAP summary dot plot. LBXVIDMS: Serum vitamin D; DEQ038Q: sunburn times; RIDAGEYR: age; RIAGENDR: gender (1=male, 2=female); RIDRETH1: race (1=Mexican American, 2=Other Hispanic, 3=Non-Hispanic White, 4=Non-Hispanic Black, 5=Other Race - Including Multi-Racial), DMDEDUC2: education (1=Less Than 9th Grade, 2 = 9-11th Grade, 3=High School Grad/GED or Equivalent, 4=Some college or AA degree, 5=College Graduate or above); DMDMARTL: marriage (1=Married, 2=Widowed, 3=Divorced, 4=Separated, 5=Never married, 6=Living with partner); BMIQ: BMI(1=normal (25<), 2=overweight (25≤BMI<30), 3=obesity (≥30)); SMQ020: smoking (1= No, 2= Yes); ALQ151: alcohol (1= No, 2= Yes); DIQ010: diabetes (1= No, 2= Yes, 3=Borderline, 9=Missing); HYTQ (1= No, 2= Yes).

Due to the difficulty of clinical acceptance of prediction models that could not be directly explained and interpreted, the SHAP method was used to explain the output of the final model by calculating the contribution of each variable to the prediction. This interpretable approach provided two types of explanations: feature-level model global explanations and individual-level local explanations. Global explanations described the overall functionality of the model. As shown in the SHAP summary plots (**Figures 5B, D**), the contribution of features to the model was evaluated using average SHAP values and displayed in descending order.

Furthermore, local explanations analyzed how specific predictions are made for individual cases by combining personalized input data. According to the prediction model, **Figure 4A** represented the patient with a probability of 0.209% towards the “cutaneous melanoma” class, while the patient with a probability of 99.8% towards the “non-cutaneous melanoma “ class. The actual measured values of the features were also shown in the waterfall plot, as depicted in **Figure 5A**. As observed, the values of LBXVIDMS, INDFMPIR, RIDAGEYR, DMDMARTL, BMIQ, DIQ010, RIDRETH1 and 4 other features would contribute to the categorization as “non-cutaneous melanoma”. If the actual values of most features were within the normal range, such as LBXVIDMS, the risk of “cutaneous melanoma” would be low. On the other hand, if the actual value of LBXVIDMS went beyond the normal range, it might increase the risk of “cutaneous melanoma” for the patient, even if the overall prediction put the case into the “non-cutaneous melanoma” class.

**Figure 6:**
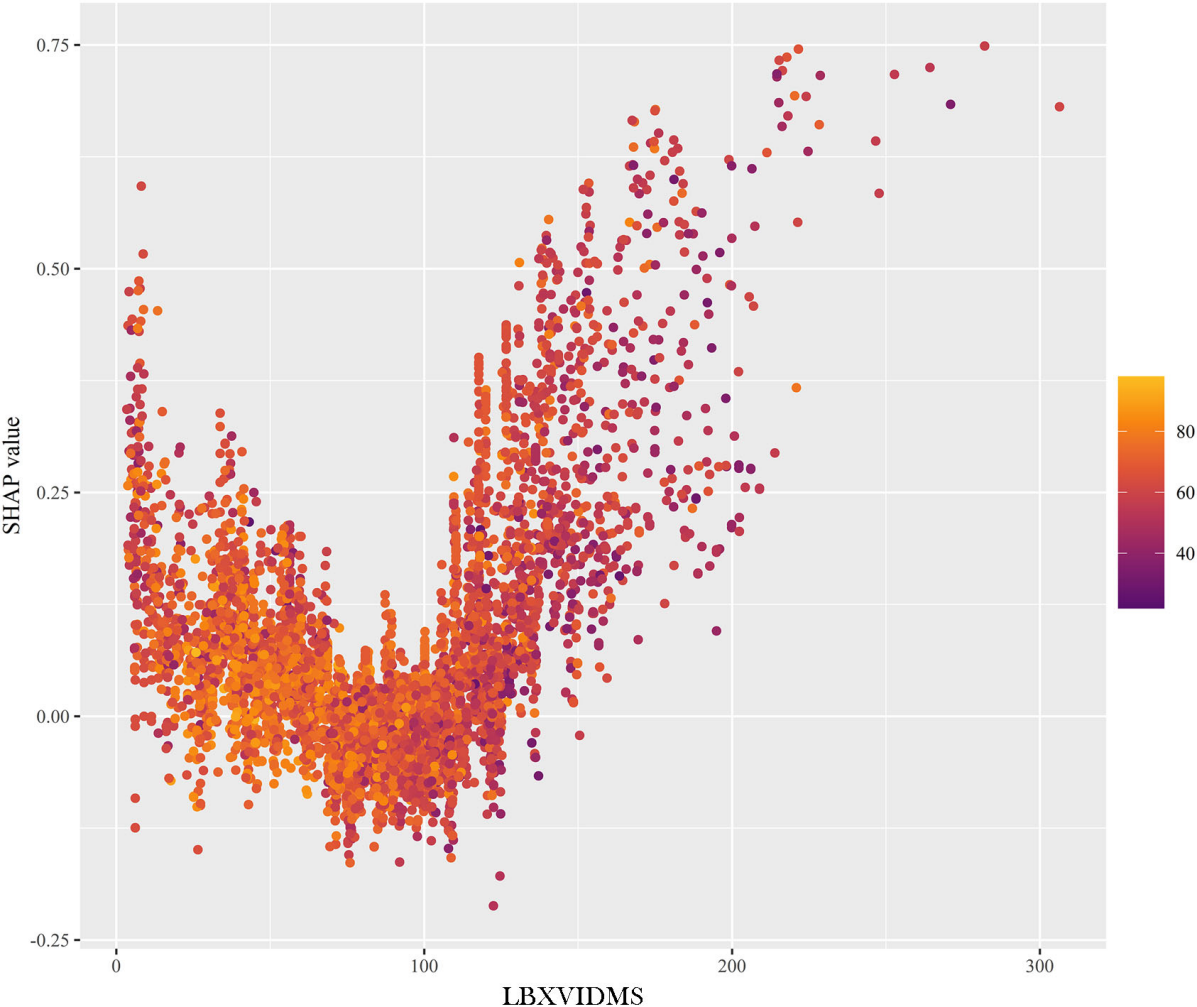
Partial dependence plot. LBXVIDMS: Serum vitamin D.

In **Figure 5C**, a similar phenomenon was observed for patients who experienced “non-cutaneous melanoma”. The features that drove the decision towards or away from the “non-cutaneous melanoma” class and their actual measurement values were shown in **Figure 5C**. Additionally, **Figure 5C** illustrated the explanatory force plots for patients in the internal validation cohort. **Figure 5C** represented cases where the likelihood of “cutaneous melanoma” was lower, while the likelihood of “non-cutaneous melanoma” was higher. At last, we conducted a partial dependence plot to detect the key feature (vitaminD) impact on SHAP value (**Figure 6**).

## Discussion

In this study, we found that vitamin D (Q2 (OR=0.97, 95% CI (0.44, 0.98)), Q3 (OR=0.71, 95% CI (0.65, 0.92)), and Q4 (OR=0.32, 95% CI (0.55, 0.81)), sunburn, advanced age, Caucasian, educational attainment some college, single and unmarried, smoking, diabetes and hypertension may all influence the development of CM. Further subgroup analyzes found that vitamin D had a protective effect against CM especially in female (OR=0.29 (95% CI: 0.15–0.45)) and young people (OR=0.74, 95% CI (0.41, 0.98)). Sun exposure is known to promote the synthesis of vitamin D, also known as the “sunshine vitamin”, which is a hormone proponent (15). Previous studies demonstrate that lack of vitamin D has been linked to an increased risk of several cancers, including CM (16, 17). This can be explained by how vitamin D inhibits the proliferation of CM cells both *in vitro* and *in vivo*. There are currently no established guidelines for measuring serum vitamin D levels or recommending vitamin D supplementation for patients with CM (18, 19). Although exposure to sunlight may increase the availability of vitamin D, clinicians should stress that adequate serum concentrations are typically achieved through dietary intake of the vitamin (20–23). Because there is a risk of heterochronic skin cancers in CM patients with vitamin D deficiency, oral vitamin D supplements should be chosen over unprotected sun exposure (24).

Additionally, we carried out an age and gender subgroup analysis and discovered that men were more likely to develop CM than women. Older people had a higher risk of developing CM than younger people did. Numerous studies over the past few years have focused on gender differences in cutaneous CM, and the findings indicate that compared to men (25, 26). Independent of the primary histological and clinical prognostic factors, women have a higher survival rate. The relative excess risk of death in men was found to be 2.70 (95% CI 2.38-3.06) in a retrospective study involving 10,538 CM patients, with survival rates remaining higher in women after accounting for numerous confounding factors (26).

Age-related effects on CM risk factors, prognosis, SLN positivity, and BRAF mutations have been supported by some studies (27–29). Offering treatment options takes age into account as well. The impact of CM drugs on frequent hospital visits for regular infusions and imaging, as well as toxicity, needs to be carefully assessed, especially in older patients with limited social support. Immunotherapy and targeted therapies give similar responses and toxicity to older patients, despite more non-CM skin cancers on targeted therapies (30, 31).

A previous meta-analysis highlighted the potential of machine learning in melanoma screening, categorizing 48 studies into seven groups based on algorithmic approaches (33). These groups included: artificial neural network (n=23), support vector machines (n=8), decision tree (n=5), cluster analysis (n=4), Bayesian network (n=3) and other (n=10). Analysis techniques used for the papers in the “other” group comprised border analysis, k-nearest neighbor, forest plot, principal component analysis, low level image processing, and unique algorithms. The mean sensitivity and specificity for each group were calculated. The mean sensitivity and specificity of the 48 articles are 87.6 and 83.54. Another recent meta-analysis including 30 references revealed prediction of immunotherapy response and prognosis of melanoma (34). The pooled AUC was 0.728 (95%CI: 0.629–0.828) for PFS in the training set, 0.760 (95%CI: 0.728–0.792) and 0.819 (95%CI: 0.757–0.880) for treatment response in the training and validation sets, respectively, and 0.746 (95%CI: 0.721–0.771) and 0.700 (95%CI: 0.677–0.724) for OS in the training and validation sets, respectively. In this study, we trained an XGBoost model for melanoma screening, achieving a high AUC of 0.906. Compared to existing approaches, representing a substantial advancement in melanoma screening technology. Unlike most published studies focusing on prognostic prediction (32–34), our work provides both clinically actionable risk factor analysis and an advanced screening tool, bridging the gap between epidemiological research and clinical application in melanoma detection and prevention. Most of the published articles focus on the prognosis prediction of melanoma using informatics, while our study is based on a large scale population level data, which is a clinically practical and advanced (35, 36).

## Conclusion

This study identifies key risk factors for cutaneous melanoma (CM), including vitamin D levels (Q2-Q4), sunburn, advanced age, Caucasian ethnicity, educational attainment, marital status, smoking, diabetes, and hypertension, with vitamin D showing a protective effect, particularly in females and younger individuals. Subgroup analysis revealed higher CM risk in men and older individuals, consistent with prior research. The XGBoost model achieved superior predictive performance (AUC = 0.906) for CM screening, outperforming other algorithms like KNN and SVM, while the SHAP method provided interpretable insights into feature contributions, enhancing clinical applicability. Bridging epidemiological research and clinical practice, the study offers actionable risk factor analysis and an advanced screening tool, emphasizing the importance of vitamin D supplementation over unprotected sun exposure and the need for age- and gender-specific prevention strategies.

## Strength and limitations

This study had key strengths as below: firstly, comprehensive multi-covariate adjustments (e.g., demographics, lifestyle, comorbidities) to isolate vitamin D’s protective effect against melanoma; secondly, rigorous model comparison across ten ML algorithms, with XGBoost achieving exceptional predictive accuracy (AUC=0.906); thirdly, interpretable AI via SHAP values, clearly identifying vitamin D as the top contributor to predictions. However, there are still some limitations of this study. Firstly, the data used in this study were from a cross-sectional study and the analysis performed could not explain the causal relationship of CM. Secondly, the inclusion of covariates in this study may still be inadequate and may overlook the effect of other factors on CM. Thirdly, the sample period of this study was 6 years across and there may be bias in the data measurement due to time variation. Finally, CM, the target of analysis in this study, is a relatively rare disease and the sample of CM patients included in this study was limited.

## Future recommendation

To advance the findings of this study, future research should prioritize longitudinal cohorts to establish causality between vitamin D and melanoma risk, addressing the limitations of cross-sectional data. Expanding sample diversity and integrating multi-omics data could uncover mechanistic pathways and improve generalizability. Enhancing predictive models via hybrid approaches or dynamic feature engineering may refine accuracy.

## Data Availability

The original contributions presented in the study are included in the article/supplementary material, Further inquiries can be directed to the corresponding author/s.
